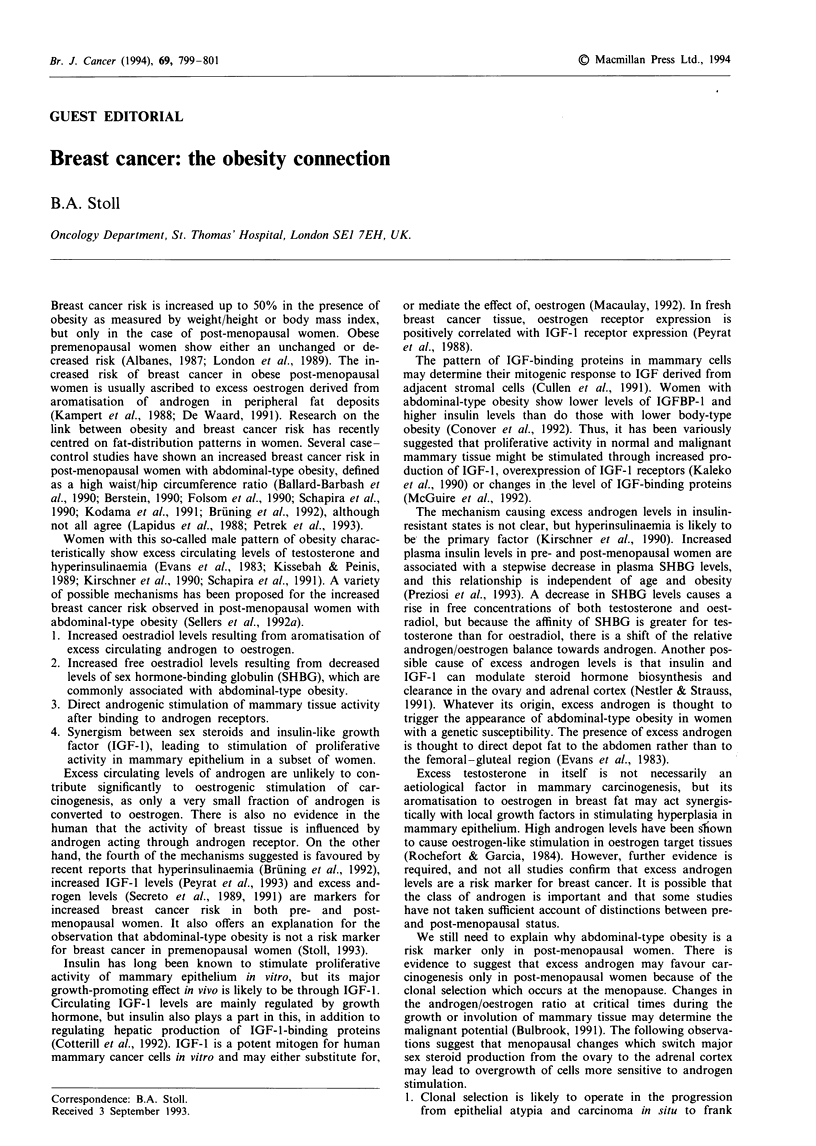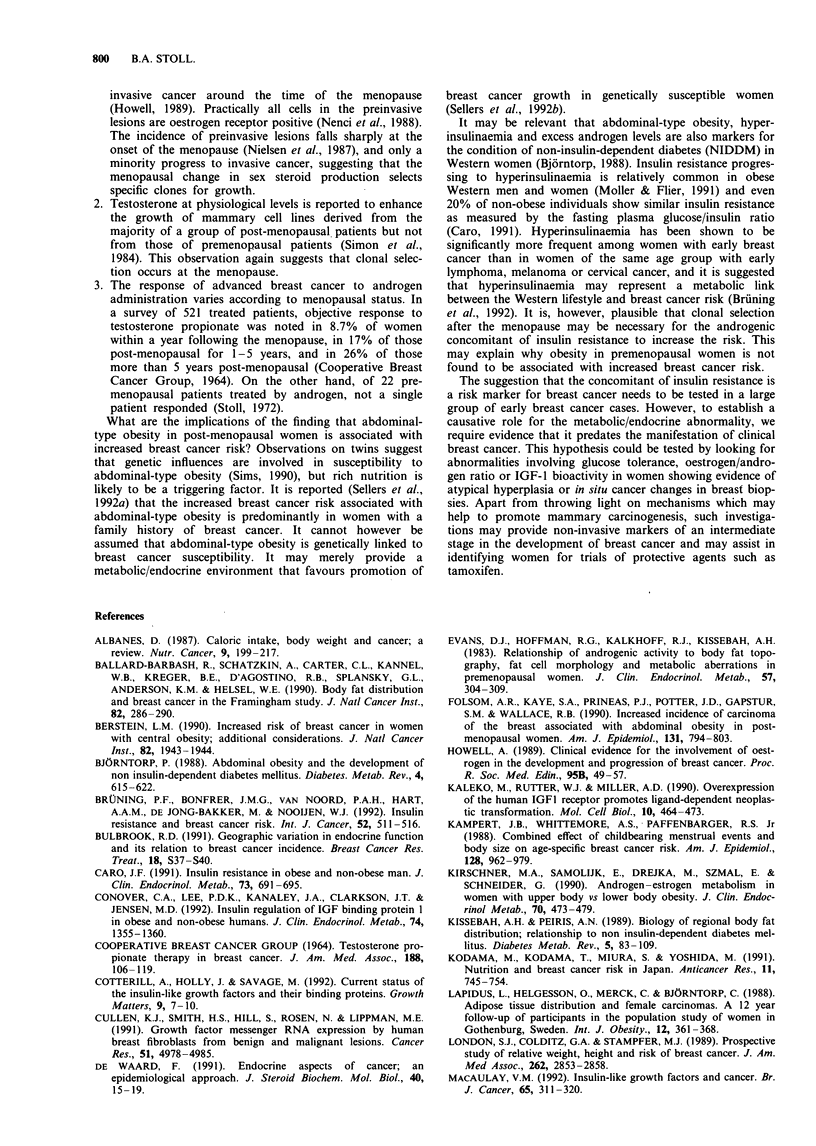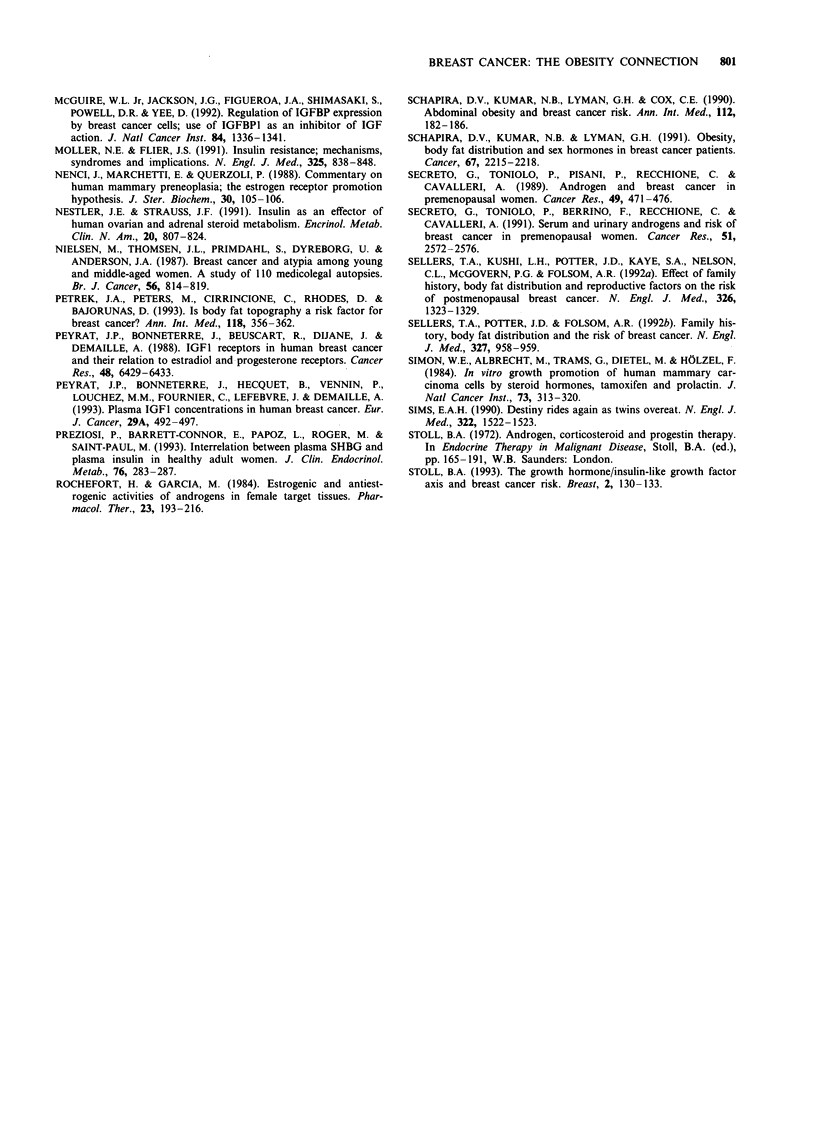# Breast cancer: the obesity connection.

**DOI:** 10.1038/bjc.1994.157

**Published:** 1994-05

**Authors:** B. A. Stoll


Br. J. Cancer (1994), 69, 799–801 © Macmillan Press Ltd., 1994

GUEST EDITORIAL

### Breast cancer: the obesity connection

B.A. Stoll

*Oncology Department, St. Thomas' Hospital, London SE1 7EH, UK.*

Breast cancer risk is increased up to 50% in the presence of obesity as measured by weight/height or body mass index, but only in the case of post-menopausal women. Obese premenopausal women show either an unchanged or decreased risk (Albanes, 1987; London *et al.*, 1989). The increased risk of breast cancer in obese post-menopausal women is usually ascribed to excess oestrogen derived from aromatisation of androgen in peripheral fat deposits (Kampert *et al.*, 1988; De Waard, 1991). Research on the link between obesity and breast cancer risk has recently centred on fat-distribution patterns in women. Several case–control studies have shown an increased breast cancer risk in post-menopausal women with abdominal-type obesity, defined as a high waist/hip circumference ratio (Ballard-Barbash *et al.*, 1990; Berstein, 1990; Folsom *et al.*, 1990; Schapira *et al.*, 1990; Kodama *et al.*, 1991; Brüning *et al.*, 1992), although not all agree (Lapidus *et al.*, 1988; Petrek *et al.*, 1993).

Women with this so-called male pattern of obesity characteristically show excess circulating levels of testosterone and hyperinsulinaemia (Evans *et al.*, 1983; Kissebah & Peinis, 1989; Kirschner *et al.*, 1990; Schapira *et al.*, 1991). A variety of possible mechanisms has been proposed for the increased breast cancer risk observed in post-menopausal women with abdominal-type obesity (Sellers *et al.*, 1992*a*).

1. Increased oestradiol levels resulting from aromatisation of excess circulating androgen to oestrogen.
2. Increased free oestradiol levels resulting from decreased levels of sex hormone-binding globulin (SHBG), which are commonly associated with abdominal-type obesity.
3. Direct androgenic stimulation of mammary tissue activity after binding to androgen receptors.
4. Synergism between sex steroids and insulin-like growth factor (IGF-1), leading to stimulation of proliferative activity in mammary epithelium in a subset of women.

Excess circulating levels of androgen are unlikely to contribute significantly to oestrogenic stimulation of carcinogenesis, as only a very small fraction of androgen is converted to oestrogen. There is also no evidence in the human that the activity of breast tissue is influenced by androgen acting through androgen receptor. On the other hand, the fourth of the mechanisms suggested is favoured by recent reports that hyperinsulinaemia (Brüning *et al.*, 1992), increased IGF-1 levels (Peyrat *et al.*, 1993) and excess androgen levels (Secreto *et al.*, 1989, 1991) are markers for increased breast cancer risk in both pre- and post-menopausal women. It also offers an explanation for the observation that abdominal-type obesity is not a risk marker for breast cancer in premenopausal women (Stoll, 1993).

Insulin has long been known to stimulate proliferative activity of mammary epithelium *in vitro*, but its major growth-promoting effect *in vivo* is likely to be through IGF-1. Circulating IGF-1 levels are mainly regulated by growth hormone, but insulin also plays a part in this, in addition to regulating hepatic production of IGF-1-binding proteins (Cotterill *et al.*, 1992). IGF-1 is a potent mitogen for human mammary cancer cells *in vitro* and may either substitute for, or mediate the effect of, oestrogen (Macaulay, 1992). In fresh breast cancer tissue, oestrogen receptor expression is positively correlated with IGF-1 receptor expression (Peyrat *et al.*, 1988).

The pattern of IGF-binding proteins in mammary cells may determine their mitogenic response to IGF derived from adjacent stromal cells (Cullen *et al.*, 1991). Women with abdominal-type obesity show lower levels of IGFBP-1 and higher insulin levels than do those with lower body-type obesity (Conover *et al.*, 1992). Thus, it has been variously suggested that proliferative activity in normal and malignant mammary tissue might be stimulated through increased production of IGF-1, overexpression of IGF-1 receptors (Kaleko *et al.*, 1990) or changes in the level of IGF-binding proteins (McGuire *et al.*, 1992).

The mechanism causing excess androgen levels in insulin-resistant states is not clear, but hyperinsulinaemia is likely to be the primary factor (Kirschner *et al.*, 1990). Increased plasma insulin levels in pre- and post-menopausal women are associated with a stepwise decrease in plasma SHBG levels, and this relationship is independent of age and obesity (Preziosi *et al.*, 1993). A decrease in SHBG levels causes a rise in free concentrations of both testosterone and oestradiol, but because the affinity of SHBG is greater for testosterone than for oestradiol, there is a shift of the relative androgen/oestrogen balance towards androgen. Another possible cause of excess androgen levels is that insulin and IGF-1 can modulate steroid hormone biosynthesis and clearance in the ovary and adrenal cortex (Nestler & Strauss, 1991). Whatever its origin, excess androgen is thought to trigger the appearance of abdominal-type obesity in women with a genetic susceptibility. The presence of excess androgen is thought to direct depot fat to the abdomen rather than to the femoral–gluteal region (Evans *et al.*, 1983).

Excess testosterone in itself is not necessarily an aetiological factor in mammary carcinogenesis, but its aromatisation to oestrogen in breast fat may act synergistically with local growth factors in stimulating hyperplasia in mammary epithelium. High androgen levels have been shown to cause oestrogen-like stimulation in oestrogen target tissues (Rochefort & Garcia, 1984). However, further evidence is required, and not all studies confirm that excess androgen levels are a risk marker for breast cancer. It is possible that the class of androgen is important and that some studies have not taken sufficient account of distinctions between pre- and post-menopausal status.

We still need to explain why abdominal-type obesity is a risk marker only in post-menopausal women. There is evidence to suggest that excess androgen may favour carcinogenesis only in post-menopausal women because of the clonal selection which occurs at the menopause. Changes in the androgen/oestrogen ratio at critical times during the growth or involution of mammary tissue may determine the malignant potential (Bulbrook, 1991). The following observations suggest that menopausal changes which switch major sex steroid production from the ovary to the adrenal cortex may lead to overgrowth of cells more sensitive to androgen stimulation.

1. Clonal selection is likely to operate in the progression from epithelial atypia and carcinoma *in situ* to frank

Correspondence: B.A. Stoll.
Received 3 September 1993.

invasive cancer around the time of the menopause (Howell, 1989). Practically all cells in the preinvasive lesions are oestrogen receptor positive (Nenci *et al.*, 1988). The incidence of preinvasive lesions falls sharply at the onset of the menopause (Nielsen *et al.*, 1987), and only a minority progress to invasive cancer, suggesting that the menopausal change in sex steroid production selects specific clones for growth.

2. Testosterone at physiological levels is reported to enhance the growth of mammary cell lines derived from the majority of a group of post-menopausal patients but not from those of premenopausal patients (Simon *et al.*, 1984). This observation again suggests that clonal selection occurs at the menopause.
3. The response of advanced breast cancer to androgen administration varies according to menopausal status. In a survey of 521 treated patients, objective response to testosterone propionate was noted in 8.7% of women within a year following the menopause, in 17% of those post-menopausal for 1–5 years, and in 26% of those more than 5 years post-menopausal (Cooperative Breast Cancer Group, 1964). On the other hand, of 22 premenopausal patients treated by androgen, not a single patient responded (Stoll, 1972).

What are the implications of the finding that abdominal-type obesity in post-menopausal women is associated with increased breast cancer risk? Observations on twins suggest that genetic influences are involved in susceptibility to abdominal-type obesity (Sims, 1990), but rich nutrition is likely to be a triggering factor. It is reported (Sellers *et al.*, 1992*a*) that the increased breast cancer risk associated with abdominal-type obesity is predominantly in women with a family history of breast cancer. It cannot however be assumed that abdominal-type obesity is genetically linked to breast cancer susceptibility. It may merely provide a metabolic/endocrine environment that favours promotion of breast cancer growth in genetically susceptible women (Sellers *et al.*, 1992*b*).

It may be relevant that abdominal-type obesity, hyperinsulinaemia and excess androgen levels are also markers for the condition of non-insulin-dependent diabetes (NIDDM) in Western women (Björntorp, 1988). Insulin resistance progressing to hyperinsulinaemia is relatively common in obese Western men and women (Moller & Flier, 1991) and even 20% of non-obese individuals show similar insulin resistance as measured by the fasting plasma glucose/insulin ratio (Caro, 1991). Hyperinsulinaemia has been shown to be significantly more frequent among women with early breast cancer than in women of the same age group with early lymphoma, melanoma or cervical cancer, and it is suggested that hyperinsulinaemia may represent a metabolic link between the Western lifestyle and breast cancer risk (Brüning *et al.*, 1992). It is, however, plausible that clonal selection after the menopause may be necessary for the androgenic concomitant of insulin resistance to increase the risk. This may explain why obesity in premenopausal women is not found to be associated with increased breast cancer risk.

The suggestion that the concomitant of insulin resistance is a risk marker for breast cancer needs to be tested in a large group of early breast cancer cases. However, to establish a causative role for the metabolic/endocrine abnormality, we require evidence that it predates the manifestation of clinical breast cancer. This hypothesis could be tested by looking for abnormalities involving glucose tolerance, oestrogen/androgen ratio or IGF-1 bioactivity in women showing evidence of atypical hyperplasia or *in situ* cancer changes in breast biopsies. Apart from throwing light on mechanisms which may help to promote mammary carcinogenesis, such investigations may provide non-invasive markers of an intermediate stage in the development of breast cancer and may assist in identifying women for trials of protective agents such as tamoxifen.

### References

ALBANES, D. (1987). Caloric intake, body weight and cancer; a review. *Nutr. Cancer*, **9**, 199–217.

BALLARD-BARBASH, R., SCHATZKIN, A., CARTER, C.L., KANNEL, W.B., KREGER, B.E., D'AGOSTINO, R.B., SPLANSKY, G.L., ANDERSON, K.M. & HELSEL, W.E. (1990). Body fat distribution and breast cancer in the Framingham study. *J. Natl Cancer Inst.*, **82**, 286–290.

BERSTEIN, L.M. (1990). Increased risk of breast cancer in women with central obesity; additional considerations. *J. Natl Cancer Inst.*, **82**, 1943–1944.

BJÖRNTORP, P. (1988). Abdominal obesity and the development of non insulin-dependent diabetes mellitus. *Diabetes. Metab. Rev.*, **4**, 615–622.

BRÜNING, P.F., BONFRER, J.M.G., VAN NOORD, P.A.H., HART, A.A.M., DE JONG-BAKKER, M. & NOOIJEN, W.J. (1992). Insulin resistance and breast cancer risk. *Int. J. Cancer*, **52**, 511–516.

BULBROOK, R.D. (1991). Geographic variation in endocrine function and its relation to breast cancer incidence. *Breast Cancer Res. Treat.*, **18**, S37–S40.

CARO, J.F. (1991). Insulin resistance in obese and non-obese man. *J. Clin. Endocrinol. Metab.*, **73**, 691–695.

CONOVER, C.A., LEE, P.D.K., KANALEY, J.A., CLARKSON, J.T. & JENSEN, M.D. (1992). Insulin regulation of IGF binding protein 1 in obese and non-obese humans. *J. Clin. Endocrinol. Metab.*, **74**, 1355–1360.

COOPERATIVE BREAST CANCER GROUP (1964). Testosterone propionate therapy in breast cancer. *J. Am. Med. Assoc.*, **188**, 106–119.

COTTERILL, A., HOLLY, J. & SAVAGE, M. (1992). Current status of the insulin-like growth factors and their binding proteins. *Growth Matters*, **9**, 7–10.

CULLEN, K.J., SMITH, H.S., HILL, S., ROSEN, N. & LIPPMAN, M.E. (1991). Growth factor messenger RNA expression by human breast fibroblasts from benign and malignant lesions. *Cancer Res.*, **51**, 4978–4985.

DE WAARD, F. (1991). Endocrine aspects of cancer; an epidemiological approach. *J. Steroid Biochem. Mol. Biol.*, **40**, 15–19.

EVANS, D.J., HOFFMAN, R.G., KALKHOFF, R.J., KISSEBAH, A.H. (1983). Relationship of androgenic activity to body fat topography, fat cell morphology and metabolic aberrations in premenopausal women. *J. Clin. Endocrinol. Metab.*, **57**, 304–309.

FOLSOM, A.R., KAYE, S.A., PRINEAS, P.J., POTTER, J.D., GAPSTUR, S.M. & WALLACE, R.B. (1990). Increased incidence of carcinoma of the breast associated with abdominal obesity in post-menopausal women. *Am. J. Epidemiol.*, **131**, 794–803.

HOWELL, A. (1989). Clinical evidence for the involvement of oestrogen in the development and progression of breast cancer. *Proc. R. Soc. Med. Edin.*, **95B**, 49–57.

KALEKO, M., RUTTER, W.J. & MILLER, A.D. (1990). Overexpression of the human IGF1 receptor promotes ligand-dependent neoplastic transformation. *Mol. Cell Biol.*, **10**, 464–473.

KAMPERT, J.B., WHITTEMORE, A.S., PAFFENBARGER, R.S. Jr (1988). Combined effect of childbearing menstrual events and body size on age-specific breast cancer risk. *Am. J. Epidemiol.*, **128**, 962–979.

KIRSCHNER, M.A., SAMOLIJK, E., DREJKA, M., SZMAL, E. & SCHNEIDER, G. (1990). Androgen–estrogen metabolism in women with upper body *vs* lower body obesity. *J. Clin. Endocrinol Metab.*, **70**, 473–479.

KISSEBAH, A.H. & PEIRIS, A.N. (1989). Biology of regional body fat distribution; relationship to non insulin-dependent diabetes mellitus. *Diabetes Metab. Rev.*, **5**, 83–109.

KODAMA, M., KODAMA, T., MIURA, S. & YOSHIDA, M. (1991). Nutrition and breast cancer risk in Japan. *Anticancer Res.*, **11**, 745–754.

LAPIDUS, L., HELGESSON, O., MERCK, C. & BJÖRNTORP, C. (1988). Adipose tissue distribution and female carcinomas. A 12 year follow-up of participants in the population study of women in Gothenburg, Sweden. *Int. J. Obesity.*, **12**, 361–368.

LONDON, S.J., COLDITZ, G.A. & STAMPFER, M.J. (1989). Prospective study of relative weight, height and risk of breast cancer. *J. Am. Med Assoc.*, **262**, 2853–2858.

MACAULAY, V.M. (1992). Insulin-like growth factors and cancer. *Br. J. Cancer*, **65**, 311–320.

McGUIRE, W.L. Jr, JACKSON, J.G., FIGUEROA, J.A., SHIMASAKI, S., POWELL, D.R. & YEE, D. (1992). Regulation of IGFBP expression by breast cancer cells; use of IGFBP1 as an inhibitor of IGF action. *J. Natl Cancer Inst.* **84**, 1336–1341.

MOLLER, N.E. & FLIER, J.S. (1991). Insulin resistance; mechanisms, syndromes and implications. *N. Engl. J. Med.*, **325**, 838–848.

NENCI, J., MARCHETTI, E. & QUERZOLI, P. (1988). Commentary on human mammary preneoplasia; the estrogen receptor promotion hypothesis. *J. Ster. Biochem.*, **30**, 105–106.

NESTLER, J.E. & STRAUSS, J.F. (1991). Insulin as an effector of human ovarian and adrenal steroid metabolism. *Encrinol. Metab. Clin. N. Am.*, **20**, 807–824.

NIELSEN, M., THOMSEN, J.L., PRIMDAHL, S., DYREBORG, U. & ANDERSON, J.A. (1987). Breast cancer and atypia among young and middle-aged women. A study of 110 medicolegal autopsies. *Br. J. Cancer*, **56**, 814–819.

PETREK, J.A., PETERS, M., CIRRINCIONE, C., RHODES, D. & BAJORUNAS, D. (1993). Is body fat topography a risk factor for breast cancer? *Ann. Int. Med.*, **118**, 356–362.

PEYRAT, J.P., BONNETERRE, J., BEUSCART, R., DIJANE, J. & DEMAILLE, A. (1988). IGF1 receptors in human breast cancer and their relation to estradiol and progesterone receptors. *Cancer Res.*, **48**, 6429–6433.

PEYRAT, J.P., BONNETERRE, J., HECQUET, B., VENNIN, P., LOUCHEZ, M.M., FOURNIER, C., LEFEBVRE, J. & DEMAILLE, A. (1993). Plasma IGF1 concentrations in human breast cancer. *Eur. J. Cancer*, **29A**, 492–497.

PREZIOSI, P., BARRETT-CONNOR, E., PAPOZ, L., ROGER, M. & SAINT-PAUL, M. (1993). Interrelation between plasma SHBG and plasma insulin in healthy adult women. *J. Clin. Endocrinol. Metab.*, **76**, 283–287.

ROCHEFORT, H. & GARCIA, M. (1984). Estrogenic and antiestrogenic activities of androgens in female target tissues. *Pharmacol. Ther.*, **23**, 193–216.

SCHAPIRA, D.V., KUMAR, N.B., LYMAN, G.H. & COX, C.E. (1990). Abdominal obesity and breast cancer risk. *Ann. Int. Med.*, **112**, 182–186.

SCHAPIRA, D.V., KUMAR, N.B. & LYMAN, G.H. (1991). Obesity, body fat distribution and sex hormones in breast cancer patients. *Cancer*, **67**, 2215–2218.

SECRETO, G., TONIOLO, P., PISANI, P., RECCHIONE, C. & CAVALLERI, A. (1989). Androgen and breast cancer in premenopausal women. *Cancer Res.*, **49**, 471–476.

SECRETO, G., TONIOLO, P., BERRINO, F., RECCHIONE, C. & CAVALLERI, A. (1991). Serum and urinary androgens and risk of breast cancer in premenopausal women. *Cancer Res.*, **51**, 2572–2576.

SELLERS, T.A., KUSHI, L.H., POTTER, J.D., KAYE, S.A., NELSON, C.L., McGOVERN, P.G. & FOLSOM, A.R. (1992*a*). Effect of family history, body fat distribution and reproductive factors on the risk of postmenopausal breast cancer. *N. Engl. J. Med.*, **326**, 1323–1329.

SELLERS, T.A., POTTER, J.D. & FOLSOM, A.R. (1992*b*). Family history, body fat distribution and the risk of breast cancer. *N. Engl. J. Med.*, **327**, 958–959.

SIMON, W.E., ALBRECHT, M., TRAMS, G., DIETEL, M. & HÖLZEL, F. (1984). *In vitro* growth promotion of human mammary carcinoma cells by steroid hormones, tamoxifen and prolactin. *J. Natl Cancer Inst.*, **73**, 313–320.

SIMS, E.A.H. (1990). Destiny rides again as twins overeat. *N. Engl. J. Med.*, **322**, 1522–1523.

STOLL, B.A. (1972). Androgen, corticosteroid and progestin therapy. In *Endocrine Therapy in Malignant Disease*, Stoll, B.A. (ed.), pp. 165–191, W.B. Saunders: London.

STOLL, B.A. (1993). The growth hormone/insulin-like growth factor axis and breast cancer risk. *Breast*, **2**, 130–133.